# 3D graph contrastive learning for molecular property prediction

**DOI:** 10.1093/bioinformatics/btad371

**Published:** 2023-06-08

**Authors:** Kisung Moon, Hyeon-Jin Im, Sunyoung Kwon

**Affiliations:** Department of Information Convergence Engineering, Pusan National University, Yangsan 50612, Korea; Department of Information Convergence Engineering, Pusan National University, Yangsan 50612, Korea; Department of Information Convergence Engineering, Pusan National University, Yangsan 50612, Korea; School of Biomedical Convergence Engineering, Pusan National University, Yangsan 50612, Korea; Center for Artificial Intelligence Research, Pusan National University, Busan 46241, Korea

## Abstract

**Motivation:**

Self-supervised learning (SSL) is a method that learns the data representation by utilizing supervision inherent in the data. This learning method is in the spotlight in the drug field, lacking annotated data due to time-consuming and expensive experiments. SSL using enormous unlabeled data has shown excellent performance for molecular property prediction, but a few issues exist. (i) Existing SSL models are large-scale; there is a limitation to implementing SSL where the computing resource is insufficient. (ii) In most cases, they do not utilize 3D structural information for molecular representation learning. The activity of a drug is closely related to the structure of the drug molecule. Nevertheless, most current models do not use 3D information or use it partially. (iii) Previous models that apply contrastive learning to molecules use the augmentation of permuting atoms and bonds. Therefore, molecules having different characteristics can be in the same positive samples. We propose a novel contrastive learning framework, small-scale 3D Graph Contrastive Learning (3DGCL) for molecular property prediction, to solve the above problems.

**Results:**

3DGCL learns the molecular representation by reflecting the molecule’s structure through the pretraining process that does not change the semantics of the drug. Using only 1128 samples for pretrain data and 0.5 million model parameters, we achieved state-of-the-art or comparable performance in six benchmark datasets. Extensive experiments demonstrate that 3D structural information based on chemical knowledge is essential to molecular representation learning for property prediction.

**Availability and implementation:**

Data and codes are available in https://github.com/moonkisung/3DGCL.

## 1 Introduction

Self-supervised learning (SSL) learns data semantics by utilizing inherent supervision in data from a large amount of unlabeled data. A self-supervised model using a pretext task has recently outperformed the general supervised model. SSL has been extremely successful in computer vision (Chen *et al.* 2020) and language fields (Mikolov *et al.* 2013, Kenton and Toutanova 2019) and has attracted particular attention in the field of drugs (Rong *et al.* 2020), wherein considerable time and money are incurred labeling data, and there is a lack of annotated data compared with other domains. A molecule can be expressed in various ways, such as a chemical fingerprint, e.g. ECFP (Rogers and Hahn 2010), which uses a fixed vector for particular substructures, and simplified molecular input line entry system (SMILES, Weininger 1988) which represents the molecule as a string. In addition, there is a way to represent a molecule as a graph, and graph neural networks (GNNs) is widely used for molecular property prediction (Gilmer *et al.* 2017) because it can reflect the structure and correlation of atoms and bonds effectively. SSL using enormous amounts of unlabeled data has shown excellent performance for molecular property prediction (Li *et al.* 2021, Subramonian 2021). However, a few issues exist. First, existing SSL models are “large-scale.” They require a million sizes of pretrain data to generalize various downstream tasks and, in many cases, are large-size models such as transformer (Vaswani *et al.* 2017) to learn that data. Therefore, there is a limitation to implementing SSL where the computing resource is insufficient. We use only 1128 samples for pretrain data, about 0.5 million model parameters, and overcome the not-high computing environment. Second, in most cases, self-supervised models do not utilize 3D structural information for molecular representation learning. The activity and property of a drug are closely related to the structure of the drug molecule. Nevertheless, most current self-supervised models do not use 3D information or use it partially (Liu *et al.* 2022a, Stärk *et al.* 2022). We introduce a novel 3D–3D view contrastive learning method to learn molecular structural-semantic. Contrastive learning is one of the SSL methods and consists of pretext tasks to learn similarities and dissimilarities between positive and negative pairs. Finally, previous models that apply contrastive learning to molecules have used the augmentation permuting atoms and bonds, while positive samples should be intrinsically identical to each other. Unlike images, molecules can be completely different if we use the augmentation that changes atoms or bonds, so molecules having different characteristics can be in the same positive samples. We generate a conformer pool consisting of several conformers to preserve the molecular composition and use it for molecule-contrastive learning.

We present a 3D Graph Contrastive Learning (3DGCL) framework for molecular property prediction, a small-scale method that uses a tiny dataset, model, and 3D coordinates. Our approach uses ∼1000 data and 0.5 million model parameters, and randomly selects molecules from the conformer pool instead of selecting the most stable molecules to learn the 3D structure abundantly. We demonstrated the effectiveness of the 3DGCL through extensive experiments. We compared the proposed method with previous state-of-the-art baselines in four regression and two classification benchmark datasets under the same experimental settings. To investigate the importance of the 3D view, we compared our method of utilizing molecular 3D coordinates with the existing pretraining method of modifying the original molecule. In addition, we also compare our conformer pool, comprising conformers that exist in nature, with molecules that are difficult to exist in nature, which is created by adding noise to the structure of the original molecule. We achieved outstanding performance and the best result in the pretraining effect by comparing the difference between pretraining and non-pretraining, compared with existing methods. The results showed that chemical-based 3D structural information is vital for molecular representation learning in property prediction. We focus not only on large-scale learning but also on pretraining strategies to learn representations correctly in SSL.

Our main contributions are as follows:

- We develop a compact SSL approach that can be run even in environments with low computational resources, using the small-scale pretrain samples and parameters. We also achieve the state-of-the-art or comparable performance in six molecular benchmarks.
- To the best of our knowledge, we propose 3D–3D view contrastive learning that can take full advantage of 3D information for the first time. We actively utilize 3D positional information inherent in molecules through a pretraining scheme using a conformer pool.
- Extensive experiments demonstrate that our method, which can utilize structural information abundantly while maintaining semantics, is more suitable for molecular property prediction than conventional contrastive learning, which may change the structure or properties of molecules.

## 2 Related works

### 2.1 Graph neural networks

GNNs are a widely used deep learning technique using graph-structured data. Due to the fact that molecules can be well described in graphs, GNNs for molecular property prediction has been active research (Gilmer *et al.* 2017, Yang *et al.* 2019, Lu *et al.* 2019). The molecular graph is represented as G=(V,E), where V and E denote the set of atoms and bonds, respectively.

The message passing scheme in GNNs (Gilmer *et al.* 2017) can be formalized as follows:


(1) $$m_{v}^{\left(k+1\right)}=\sum_{w\in N(v)}M_{t}(h_{v}^{\left(k\right)},h_{w}^{\left(k\right)},e_{vw})$$

(2) $$h_{v}^{\left(k+1\right)}=U_{k}(h_{v}^{\left(k\right)},m_{v}^{\left(k+1\right)})$$

(3) $$h_{G}=\text{READOUT}(h_{v}^{\left(k\right)}\;|\;v\in G)$$

The GNNs aims to learn each node vector $h_{v}^{k}$ and the entire graph vector $h_{G}$ in the *k*-th layers. N(v) denotes the neighbor node of node *v* and $e_{vw}$ denotes the edge between node *v* and node *w*. *M* and *U* are message and update functions depending on the GNN models. GNNs update each node through an iterative message passing process. Finally, the readout layer, e.g. sum or mean pooling, is applied to get the entire graph vector while satisfying permutation-invariance.

### 2.2 Self-supervised learning for molecular property prediction

In the drug field, it is challenging to obtain annotated data due to expensive wet-lab experiments; however, it is relatively easy to collect data without annotation. Thus, there have been many SSL approaches recently, and they have shown noticeable results for molecular property prediction.

Inspired by BERT (Kenton and Toutanova 2019), a powerful pretraining model in NLP, SMILES-BERT (Wang *et al.* 2019), ChemBERTa (Chithrananda *et al.* 2020) utilized the enormous SMILES datasets to predict molecular properties. However, SMILES is not geometry-aware because it represents molecules as string sequences. Instead of SMILES as molecular representations, recent studies using molecular graphs use various types of pretext tasks. GROVER (Rong *et al.* 2020) developed a transformer-style architecture and utilized motif-level pretext tasks. Dillard (2021) integrated pretext tasks at a different scale consisting of the atom, fragment, and molecule levels.

Contrastive learning is one of the powerful learning methods of SSL, making similar samples close and dissimilar samples far away in embedding space (He *et al.* 2020, Chen *et al.* 2020). GraphCL (You *et al.* 2020) proposed various augmentation methods for graph contrastive learning. MolCLR (Wang *et al.* 2022) focused on molecular representation learning based on approaches presented in GraphCL. These methods change the structure of the original graph, such as dropping nodes or changing edges, so that molecules can have different properties. Subramonian (2021) proposed contrastive method brings the subgraph and the graph close together through motif-level sampling from the entire graph. MoCL (Sun *et al.* 2021) used augmentation to replace the substructure in the original molecule with bioisostere, which has similar properties to the original, incorporating domain knowledge. They tried to maintain the semantic information of the molecule, but supervision used in pretraining may not be appropriate for molecular representation learning because even a tiny change in a molecule can lead to a significant difference. Hermosilla and Ropinski (2022) proposed a 3D protein contrastive learning method to learn the structure of a protein. During the pretraining process, the substructures of the protein are used, which is a similar approach to (Sun *et al.* 2021) except that 3D information is used. MEMO (Zhu *et al.* 2022) is a multiple-view contrastive learning method using various molecular representations (2D graph, 3D graph, Fingerprint, and SMILES). Uni-Mol (Zhou *et al.* 2023) is a self-supervised model that uses 3D information and consists of a pretraining scheme that predicts masked atoms or denoises 3D positions after adding noise to the molecular coordinates.

Molecular structure information plays an essential role in determining molecular properties. We have recently witnessed studies with great success using 3D geometric data. GraphMVP (Liu *et al.* 2022a) and 3Dinformax (Stärk *et al.* 2022) developed 2D–3D view contrastive learning approaches maximizing the mutual information between molecular embedding of 2D graph network and 3D graph network. Existing works with 3D coordinates do not fully utilize geometric information because they allow the 2D view network to learn 3D view information.

### 2.3 3D graph neural networks

Due to a general graph being represented in a non-euclidean space, it is difficult to grasp the exact molecular structure. However, by leveraging 3D positions, we can specifically use unique molecular geometric properties such as distance, angle, and torsion in the euclidean space. For this reason, 3D GNNs using 3D information are attracting widespread attention in drug and material discovery fields (Unke and Meuwly 2019, Qiao *et al.* 2020).

3D molecular graph can be represented as $G^{3d}=(V,E,R)$, where R denotes the 3D coordinates of atoms. SGCN (Danel *et al.* 2020) applied different weights according to interatomic distance in the message-passing process based on GCN. SchNet (Schütt *et al.* 2017) used Gaussian radial basis functions to represent distance information in a high-dimensional space. DimeNet (Gasteiger *et al.* 2020) used orthogonal functions to learn both distance and angle information. SphereNet (Liu *et al.* 2022b) also used torsion information to represent a complete molecular structure using spherical message passing. ChIRo (Adams *et al.* 2022) developed a chirality-aware 3D network that can learn molecular chirality from torsion angles. SchNet and subsequent models (DimeNet and SphereNet) can capture non-bonded interaction based on 3D positional information within cutoff. SchNet is the most efficient architecture among 3D GNNs in terms of the learning cost and time, although the other models are superior performance. We use SchNet as an encoder for 3D contrastive learning. The critical process of SchNet is expressed as follows: where $v_{i}^{0}$ denotes the feature of the atom *i* and is the initialized value using embedding of the atomic number. where $d_{ij}$ denotes the distance between the atom *i* and the atom *j*. The interatomic distance is encoded to $e_{ij}$ by radial basis functions located in *k* centers $\mu_{k}$. $v_{i}^{l+1}$ in the *l *+* *1th layer is updated based on neighbor atom *j* in the *l*th layer and the distance information $e_{ij}$. ° indicates the element-wise multiplication.


(4) $$v_{i}^{0}=a_{Z^{i}}$$

(5) $$\begin{array}{c} e_{ij}=\text{exp}\;{\left(-\gamma \left\|\begin{array}{c} d_{ij}-\mu_{k} \end{array}\right\|\right)}^{2} \end{array}$$

(6) $$\begin{array}{c} v_{i}^{\left(l+1\right)}=v_{i}^{\left(l\right)}+\text{MLP}(\sum_{j\in N(v)}v_{j}^{\left(l\right)}{}^{\circ}e_{ij}) \end{array}$$

(7) $$\begin{array}{c} h_{G^{3d}}=\sum_{i=1}^{n}\text{MLP}(v_{i}^{\left(l\right)}|v\in G) \end{array}$$

The global molecular feature vector $h_{G^{3d}}$ is obtained based on summation of the atom embeddings. We can handle arbitrary positioned atoms in the 3D space through the encoder.

## 3 Materials and methods

### 3.1 Conformer pool

**Figure 1.**
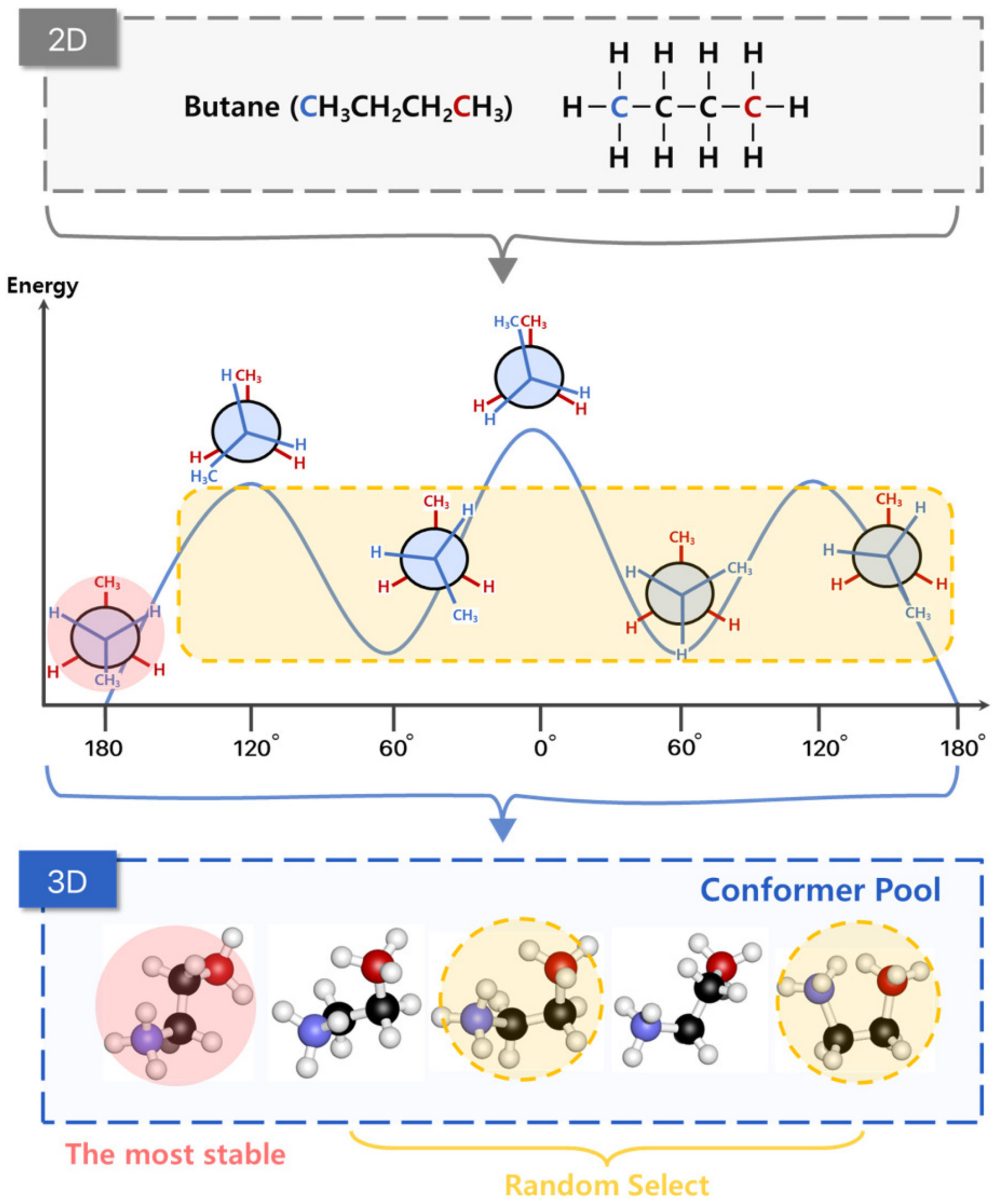
The process of creating a conformer pool we use in contrastive learning. We cannot grasp the spatial characteristics of butane in 2D. However, we can see that the potential energy varies depending on the rotation angle of the bond between the second carbon (C2) and the third carbon (C3), both colored black in the figure on top. We construct a pool of conformers with different stability and pretrain by randomly selecting from the rest of the conformers shown in the yellow box in the figure in the middle, except for the most stable molecule

A conformer is a molecule group formed by rotation on single bonds in a molecule. Conformers have different potential energies depending on the degree of rotation, and the lower the energy, the higher the probability of existence in nature. For example, butane is a molecule composed of single carbon-carbon bonds and single carbon-hydrogen bonds. If it is expressed in 2D, it is difficult to see the change due to the rotation of a single bond. Nevertheless, as shown in Fig. 1, if represented in 3D, we can see that butanes form conformers with different potential energies according to rotation. We generate a conformer pool using the Merck molecular force field (MMFF94) (Halgren 1996) function in RDkit (Landrum *et al.* 2020) to utilize diverse molecular geometric information in contrastive learning. The MMFF method combines distance geometry algorithms, a classic approach that randomly sample conformational space, with energy minimization using MMFF. The conformer pool consists of five conformers with the lowest energy. We do not add more conformers because five conformers are enough to represent almost all molecules in nature (Liu *et al.* 2022a), and the more conformers we add, the more computational cost we need. We also reduce the cost and enrich the molecular representation by randomly selecting the conformers from the conformer pool.

### 3.2 3D graph contrastive learning

Contrastive learning is a powerful SSL method, moving positive pairs of similar samples close and negative pairs of dissimilar samples far away in embedding space. In order to be consistent with the basic assumption of contrastive learning, we make the positive samples be intrinsically the same as each other, unlike previous works that have made use of the augmentation permuting atoms and bonds or may not maintain molecular semantics (Subramonian 2021, Sun *et al.* 2021, Wang *et al.* 2022).

Maintaining semantics between conformers means that conformers conserve identity with the same element. Therefore, we performed contrastive learning by constructing positive pairs with conformers (molecules with slightly different properties) instead of the existing methods of changing semantics in the pretraining process.

**Figure 2.**
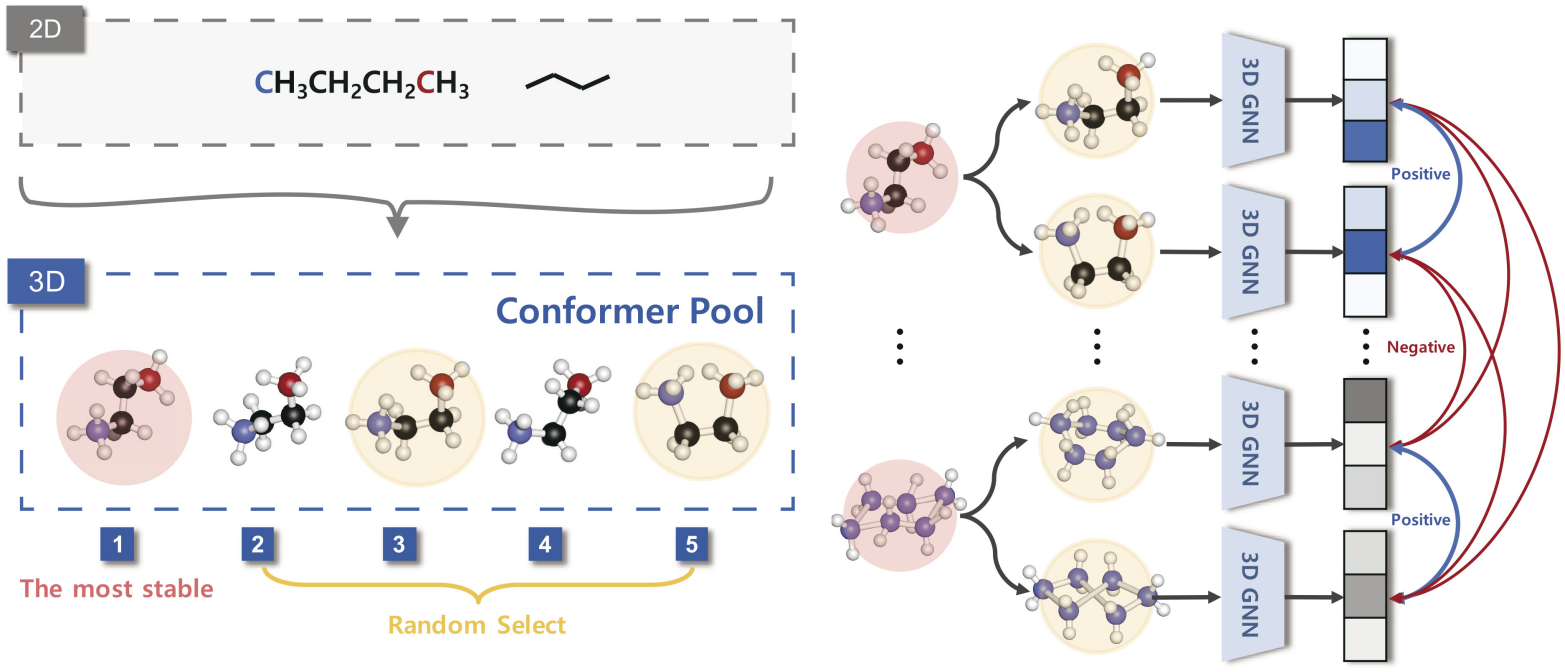
We generate a conformer pool from the original 2D molecule using 3D coordinates information. The conformer pool consists of five conformers that vary stability with the rotation angle of a single bond. We randomly choose the two molecules from the conformer pool for conformer augmentation. Then, the selected molecule is fed to a 3D encoder for embedding molecular representation. We apply a projection head to map the 3D view representations into the latent space for contrastive learning. The contrastive loss is computed across all positive and negative pairs in the minibatch of N molecules

Given molecular-input samples, we augment the molecule by applying our conformer pool to construct the positive and negative pairs. We randomly select two conformers (in yellow circle) in the conformer pool, as shown in Fig. 2.

Graph contrastive learning maximizes the consistency between latent representations of positive pairs against negative pairs. We get the molecular graph embedding *h* using a 3DGNN encoder as the SchNet, and we apply a projection head to embedding *h* resulting in the nonlinear transformation to obtain latent representation *z*. The projection head consists of a two-layer multilayer perceptron and maps the molecular representations to another latent space as advocated in (Chen *et al.* 2020). Finally, we adopt a normalized temperature-scaled cross entropy (NT-Xent) (Chen *et al.* 2020) loss as our objective loss to maximize the agreement between the positive samples compared with the negative samples in a minibatch of N molecules. The contrastive loss for the first and second sampled conformers of *k*-th graph $k_{c1},k_{c2}$ is defined as: where $\text{sim}\;(z_{1},z_{2})$ is the cosine similarity $z_{1}\cdot z_{2}/(\left\|z_{1}\right\|\left\|z_{2}\right\|)$ and τ is the temperature parameter. We compute the final loss across all training samples in the minibatch. There have been a few molecular graph contrastive learning models using diverse viewpoints recently. As introduced in the Section 2.2, MoCL and MolCLR (Sun *et al.* 2021, Wang *et al.* 2022) use the 2D–2D view approach, and there are models 3Dinformax and GraphMVP (Stärk *et al.* 2022, Liu *et al.* 2022a) that use the method of the 2D–3D view. However, as far as we know, we propose a 3D–3D perspective contrastive learning model first to fully utilize 3D information in the context of molecular property prediction, as shown in Table 1.


(8) $$\begin{array}{c} L(z_{k_{c1}},z_{k_{c2}})=-\text{log}\;\frac{\;\text{exp}(\text{sim}(z_{k_{c1}},z_{k_{c2}})/\tau )}{\sum_{l=1,l\neq k}^{2N}\;\text{exp}\;(\text{sim}(z_{k_{c1}},z_{l_{c2}})/\tau )} \end{array}$$

**Table 1.** Comparison of views in various contrastive learning models.

Model	2D–2D view	2D–3D view	3D–3D view

MoCL	✓	⋅	⋅
MolCLR	✓	⋅	⋅
3Dinformax	⋅	✓	⋅
GraphMVP	⋅	✓	⋅
**3DGCL**	⋅	⋅	✓

## 4 Experiments

### 4.1 Datasets

#### 4.1.1 Pretraining dataset

For pretraining of 3DGCL. We use only 1128 ESOL datasets (Delaney 2004). We add 3D coordinates to pretrain datasets using the Merck molecular force field (MMFF94) (Halgren 1996) function, which can obtain 3D coordinates faster (Stärk *et al.* 2022) than the latest deep learning-based methods (Ganea *et al.* 2021, Shi *et al.* 2021).

#### 4.1.2 Downstream datasets

We use four regression and two classification datasets for downstream tasks. The datasets are described in Table 2:

**Table 2.** Summary of benchmark datasets.^a^

Domain	Dataset	# Compounds	# Atoms	Metric

Physical chemistry	ESOL	1128	13.3	RMSE
	Freesolv	642	8.7	RMSE

Quantum mechanics	QM7	6834	6.8	MAE
	QM8	21786	7.8	MAE

Biophysics	BACE	1512	34.4	ROC-AUC
Physiology	BBBP	2039	24.1	ROC-AUC

a The table contains the applied domain, type, number of molecules, average number of atoms in the dataset, and metrics used.

- ESOL: water solubility data (log solubility in mols per liter) for common organic small molecules.
- Freesolv Mobley and Guthrie (2014): experimental and calculated hydration free energy of small molecules in water.
- QM7 Blum and Reymond (2009): electronic properties (atomization energy, HOMO/LUMO, etc.) determined using *ab initio* density functional theory.
- QM8 Ramakrishnan *et al.* (2015): electronic spectra and excited state energy of small molecules calculated by multiple quantum mechanic methods.
- BBBP: binary labels of blood–brain barrier penetration.
- BACE: quantitative (IC50) and qualitative (binary label) binding results for a set of inhibitors of human β-secretase 1(BACE-1).

We select the six molecule datasets from MoleculeNet (Wu *et al.* 2018). The first two, ESOL and Freesolv, are physical chemistry datasets, and the next two, QM7 and QM8, are quantum mechanic datasets, then BACE and BBBP are biophysics and physiology, respectively. We also add 3D locational information to downstream datasets in the same way (Fang *et al.* 2022) as the pretraining dataset.

### 4.2 Training and evaluation settings

#### 4.2.1 Pretraining setting

During the pretraining of the model, we use an Adam optimizer with an initial learning rate of 0.001 and exponentially reduce the learning rate at a ratio of 0.95 or 0.99. Pretraining process is run with 300 epochs and 400 batch sizes.

#### 4.2.2 Fine-tuning setting

After pretraining, we fine-tuned the model on six benchmark datasets. We train the model with an Adam optimizer of a learning rate of 0.001 and exponentially decrease the learning rate at rates of 0.95 or 0.99 in the same way as pretraining. We set 32 batch sizes and ran 200 epochs for all datasets.

We split the dataset into train/validation/test sets at a ratio of 80/10/10 using the scaffold splitter (Bemis and Murcko 1996) from DeepChem (Ramsundar *et al.* 2019) for downstream tasks like previous works (Hu *et al.* 2020, Rong *et al.* 2020). Each set is structurally different as the scaffold splitter splits molecular data by their substructure. Then, this splitting method is widely used for molecular-related tasks because it can evaluate the generalization capability of algorithms well. We provide further details of hyperparameter settings in supplemental materials.

#### 4.2.3 Implementation details

We implement our 3DGCL with PyTorch (Paszke *et al.* 2019) and PyTorch Geometric frameworks (Fey and Lenssen 2019) and write our implementation code based on (Liu *et al.* 2021). We use RDKit (Landrum *et al.* 2020), a cheminformatics software for molecular-related tasks. All the experiments are run on a single NVIDIA RTX 3090 GPU.

There are two processes regarding the running time of tasks about conformers: creating a conformer pool from the original ESOL dataset and contrastive learning after selecting two conformers. In our study, each process took 3 and 6 min, totaling ∼9 min, and the number of model parameters is ∼0.5 million. This indicates that our work requires significantly low resources.

#### 4.2.4 Evaluation

To evaluate our fine-tuned model, we measure the root mean squared error (RMSE) on ESOL, Freesolv, and the mean absolute error (MAE) on QM7 and QM8 datasets. For a fair comparison with the state-of-the-art models, we run the model with random seeds three times and average the performance and standard deviation in the same way in previous works. In the same way (Wang *et al.* 2022, Rong *et al.* 2020, Zhou *et al.* 2023), we evaluated QM7 for one target task and QM8 for the average of 12 target tasks.

### 4.3 Results

We evaluate the 3DGCL performance with standard supervised baselines and self-supervised models. All compared methods use more than one dataset of six benchmark datasets (ESOL, Freesolv, QM7, QM8, BBBP, and BACE) and conduct experiments under the same condition (Zhou *et al.* 2023). The same condition denotes scaffold-splitting (with considering chirality) the train/validation/test data to an 8:1:1 ratio and running tests independently three times with three random seeds. Scaffold splitting can be divided into two types according to the consideration of chirality. We show the results of scaffold splitting with considering chirality in Table 3, the results without considering chirality in Supplementary Table S2.

**Table 3.** Test performance of 3DGCL and other methods based on four regressions (ESOL, Freesolv, QM7, and QM8) and two classification benchmarks (BBBP and BACE).^a^

Metric	RMSE (lower is better) ↓		MAE (lower is better) ↓		ROC-AUC (higher is better) ↑	
Model	ESOL	Freesolv	QM7	QM8	BBBP	BACE

DMPNN	1.050 (0.008)	2.082 (0.082)	103.5 (8.6)	0.0190 (0.0001)	71.0(0.3)	80.9 (0.6)
Attentive FP	0.877 (0.029)	2.073 (0.183)	72.0 (2.7)	0.0179 (0.001)	64.3 (1.8)	78.4 (0.02)
HMGNN	0.832 (0.010)	1.857 (0.071)	59.0 (3.4)	0.0173 (0.004)	64.3 (1.8)	78.4 (0.02)
N-Gram_*RF*_	1.074 (0.107)	2.688 (0.085)	92.8 (4.0)	0.0236 (0.0006)	69.7 (0.6)	77.9 (1.5)
N-Gram_*XGB*_	1.083 (0.082)	5.061 (0.744)	81.9 (1.9)	0.0215 (0.0005)	69.1 (0.8)	79.1 (1.3)
PretrainGNN	1.100 (0.006)	2.764 (0.002)	113.2 (0.6)	0.0200 (0.0001)	68.7 (1.3)	84.5 (0.7)
MolCLR	1.271 (0.033)	2.594(0.249)	66.8 (2.3)	0.0178 (0.0003)	72.2 (2.1)	82.4 (0.9)
GraphMVP	1.029 (0.033)				72.4 (1.6)	81.2 (0.9)
GROVER_*base*_	0.983 (0.090)	2.176 (0.052)	94.5 (3.8)	0.0218 (0.0004)	70.0 (0.1)	82.6 (0.7)
GROVER_*large*_	0.895 (0.017)	2.272 (0.051)	92.0 (0.9)	0.0224 (0.0003)	69.5 (0.1)	81.0 (1.4)
GEM	0.798 (0.029)	1.877 (0.094)	58.9 (0.8)	0.0171 (0.0001)	72.4 (0.4)	85.6 (0.2)
Uni-Mol	0.788 (0.029)	1.620 (0.035)	**41.8** (0.2)	0.0156 (0.0001)	72.9 (0.6)	**85.7** (0.2)

**3DGCL**	**0.778 (0.102)**	**1.441** (0.19)	42.53 (7.69)	**0.0143** (0.0001)	**79.15** (0.04)	85.5 (0.03)

a We mark the best results in bold and the second best results in underlined. We split the dataset into 8:1:1 (train:validation:test) using scaffold splitting considering chirality.

The baselines are as follows: DMPNN (Yang *et al.* 2019) proposes an interactive message passing scheme considering the interactions. AttentiveFP (Xiong *et al.* 2020) is an attention-based GNN. HMGNN (Shui and Karypis 2020) utilizes global molecular representations using an attention mechanism. CD-MVGNN (Ma *et al.* 2022) performs a cross-dependent message-passing scheme considering both atom and bond information. We compare CD-MVGNN with the baselines in Supplementary Table S2 for the same test settings.

The rest of the seven methods are self-supervised models. N-Gram (Liu *et al.* 2019) produces a graph representation in short walks by building the node embedding. MolCLR (Wang *et al.* 2022) is a 2D–2D view contrastive learning model based on atom masking, bond deletion, and subgraph removal. GraphMVP (Liu *et al.* 2022a) proposes 2D–3D view contrastive learning approaches. GROVER (Rong *et al.* 2020) uses a predictive pretraining strategy of motif-level. GEM (Fang *et al.* 2022) and Uni-Mol design predictive SSL scheme using 3D molecular information. We reference the performance of the baselines in Uni-Mol (Zhou *et al.* 2023).

We present the experimental result with dataset size in pretraining to show the efficiency of our method, as shown in Table 3. We mark the best results in bold and underline the second best in Table 3. 3DGCL outperforms all other methods on four of six datasets and shows the second-best performance in QM7 and third-best in BACE. Furthermore, our method achieves overwhelming performance on Freesolv and BBBP by a large margin.

**Figure 3.**
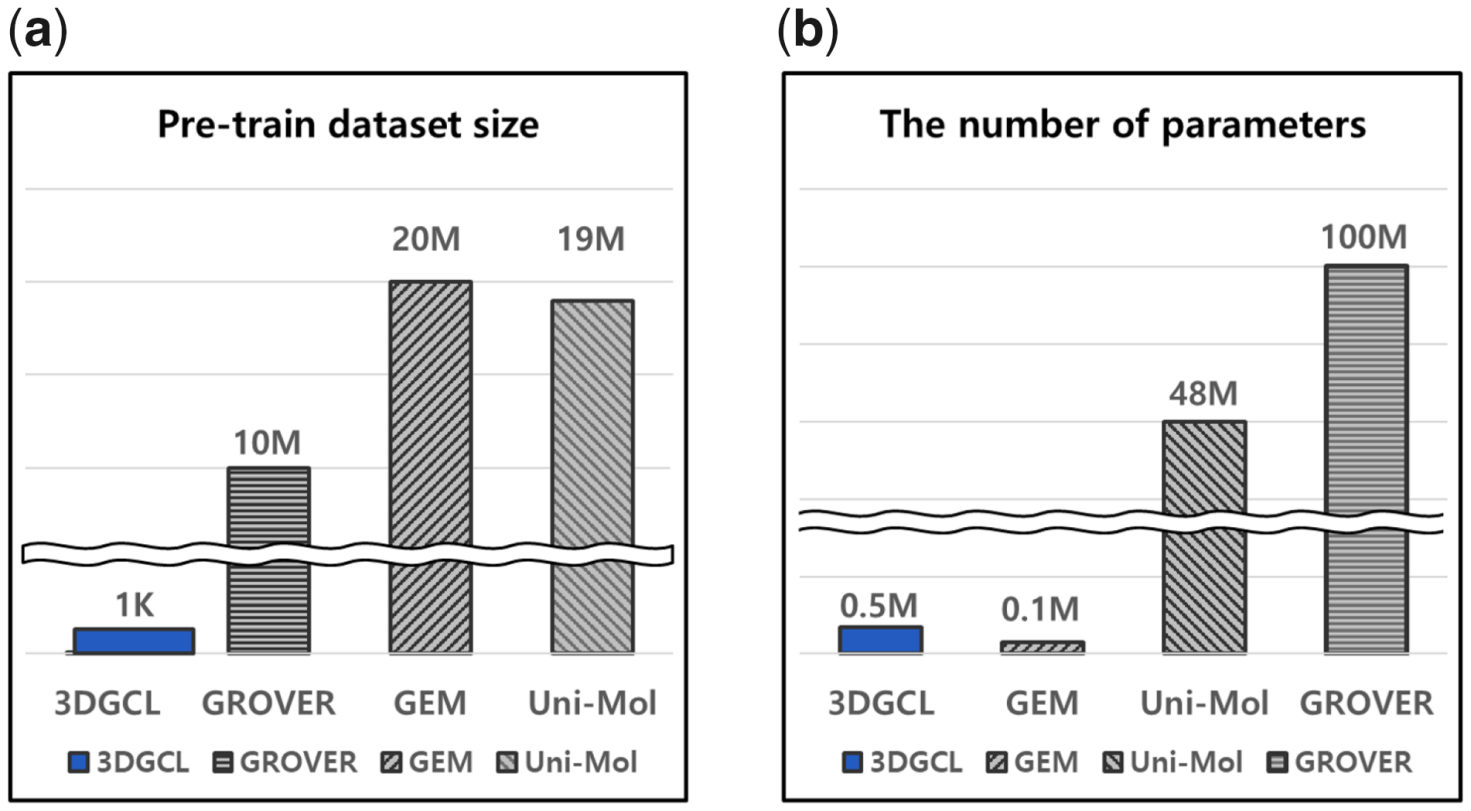
Comparison of 3DGCL and the three state-of-the-art models in terms of dataset and parameter size in pretraining

The results present that 3DGCL consistently achieves the best performance and comparable results. We should also note that our method uses overwhelmingly fewer resources than other methods, as shown in Fig. 3. We compare 3DGCL and the three state-of-the-art, GROVER, GEM, and Uni-Mol that show the best overall performance with the pretrain dataset and model parameters. We can see that 3DGCL uses about 10 000 times fewer datasets using only 1k than other state-of-the-art methods using over 10M datasets, as shown in Fig. 3a. We can also confirm that the number of 3DGCL parameters is approximately over 100 times fewer than the parameter size of other models, as shown in Fig. 3b.

## 5 Additional experiments

### 5.1 Pretraining effects according to the molecular 3D information and size

3D information is molecular coordinates, and we can obtain geometric information such as distance, angle, and torsion. Our encoder utilizes distance information in the pretraining process. To verify the contribution of geometric information on molecular representation learning, we compare our model with pretraining and the model without pretraining, i.e. SchNet. As a result of the experiment, 3DGCL consistently obtains enhanced performance on all the datasets, as shown in Table 4.

**Table 4.** Performance improvements of 3DGCL pretraining.^a^

Dataset	ESOL	Freesolv	QM7	QM8	BBBP	BACE
Molecular size	13.3	8.7	6.8	7.8	34.4	24.1


Without pretraining	1.031 (0.1)	1.91 (0.42)	59.70 (22.1)	0.0163 (0.0003)	71.19 (0.03)	76.2 (0.02)
With 3DGCL pretraining	0.778 (0.102)	1.441 (0.19)	42.53 (7.69)	0.0143 (0.0001)	79.15 (0.04)	85.5 (0.03)

**Improvement**	**24.5%**	**24.61%**	**28.8%**	**12.3%**	**10.1%**	**11%**

a We show enhanced performances to verify the pretraining effect of our method. We also indicate the number of atoms of datasets.

We also investigate the pretraining effect by molecular size. The size denotes the average number of atoms in each dataset. The result shows that 3DGCL leads to higher self-supervised performance on small molecules than on larger datasets. This confirms the effect of the backbone encoder (SchNet), which was developed to focus on small organic molecules. We demonstrate that 3D spatial information is critical for molecular property prediction, and the encoder plays an important role in SSL.

### 5.2 Comparison with diverse augmentation methods

**Figure 4.**
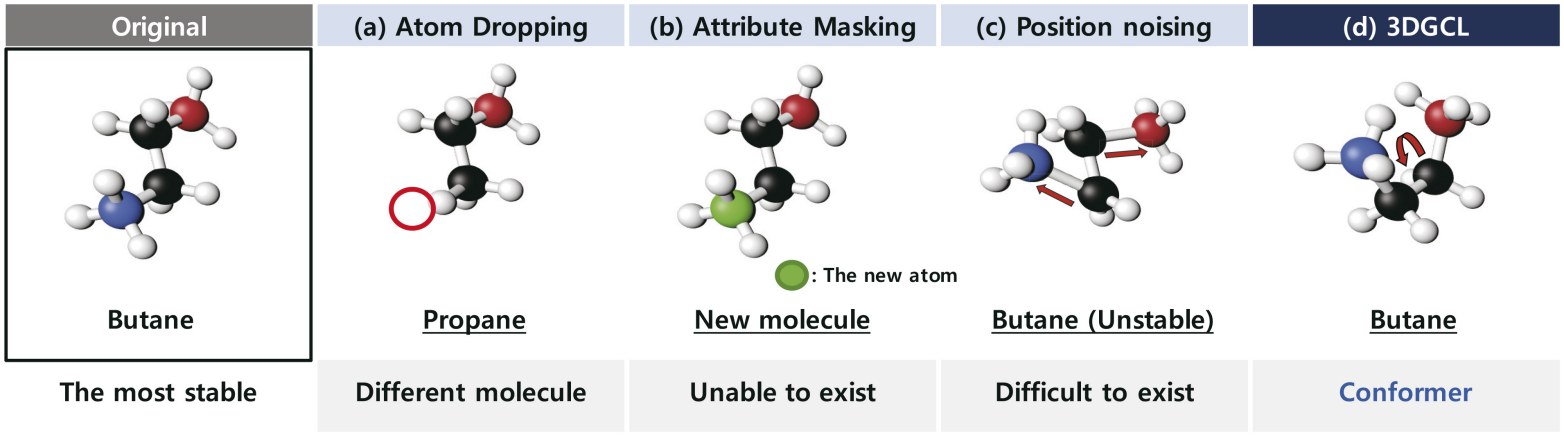
The overview of various molecular augmentation methods

We conduct comprehensive studies to compare the proposed 3DGCL method with the existing contrastive learning approaches, which may alter the original molecular properties. In addition, we also compare our conformer pool, which consists of five conformers that can most likely exist in nature, and molecules that are difficult to exist in nature, which is created by adding noise to the 3D position of the original molecule. Finally, we compared selecting the most stable molecules with our method of selecting molecules at random. We visualize the methods used in this study in Fig. 4.

**Atom dropping**: atom dropping removes atoms randomly at a constant ratio in the molecule graph G. In previous graph contrastive learning studies, atom dropping has been widely used for graph augmentation (You *et al.* 2020, Wang *et al.* 2022). We randomly delete 20% of the atoms in the original molecule.

**Attribute masking**: like atom dropping, we randomly select 20% of atoms in the original molecule, then mask their attributes with a feature not presented in the original molecules (You *et al.* 2020, Wang *et al.* 2022). Since we use only the atomic number as the feature of atoms, we replace the atom number with the new masking number *n*.

**Position noising**: we add noise to the original 3D coordinates of the most stable molecule. We generate the noise drawn from a Gaussian distribution ∼ N(0,1) whose mean and standard deviation are 0 and 1, then multiply the noise by 0.01 to fit the scale with coordinates.

**Conformer-S**: we compare the method using the two most stable conformers without creating the conformer pool to verify that the proposed way of randomly selecting in the conformer pool is effective for learning rich molecular representations. We name the approach utilizing only the two stable molecules conformer-S and call our method **Conformer-R** in this experiment.

We conduct extensive experiments to confirm the efficacy of molecular location information based on chemical knowledge. The experiments consist of three concepts. First, we compare atom dropping and atom masking methods, which are challenging to preserve molecular semantics due to severe structural changes. Second, we performed pretraining using the noising position method that utilizes 3D information while subtly giving spatial changes without chemical knowledge. Finally, we witness that our random select way is superior to the stable molecule choice method.

As experimental results, attribute masking has the least improved effect in pretraining, and atom dropping shows the second minor effect after attribute masking, as shown in Table 5. These results show that the method of significantly modifying the structure and properties of the original molecule is not suitable for molecular representation learning. Augmentation, position noising, which generates molecules that are difficult to exist (finely changing the 3D locations of atoms at random), showed a better effect than the above two approaches. We also test comparing the conformer pool (Conformer-R) and without the pool (Conformer-S) to confirm that our method plentifully learns from molecular geometric representation. As a result, our 3DGCL shows the best performance than other methods. Through diverse comparative experiments, we validate that our proposed approach is the most proper for molecular graph contrastive learning in the context of molecular property prediction.

**Table 5.** Details of the experimental results based on diverse augmentation methods.^a^

Method	ESOL	Freesolv	QM7	QM8	BBBP	BACE	Maintaining semantics	Chemical knowledge	3D information

Atom dropping	0.808	1.743	57.14	0.0164	71.68	84.61	X	X	X
Attribute masking	0.800	1.817	48.75	0.0171	75.17	84.81	X	X	X
Position noising	0.780	1.89	51.34	0.0166	77.53	84.40	X	X	O
Conformer-S	0.787	1.743	48.73	0.0155	75.82	85.34	O	O	O
**Conformer-R (ours)**	**0.778**	**1.441**	**42.53**	**0.0143**	**79.15**	**85.52**	**O**	**O**	**O**

a We conduct extensive tests to prove the superiority of our conformer-based augmentation method for molecular property prediction. The methods are categorized according to semantic preservation, domain knowledge, and 3D information, and their results are reported. We mark the best results in bold and the second best results in underline.

### 5.3 Application to real world

The drug field suffers from annotation scarcity in that wet-lab experiments are required. We design experiments to verify that our model can be applied to the actual drug area, assuming the real world with a data deficiency. We conduct the experiments using ESOL and only 150, 300, and 600 samples as training data without using the 1128 entire datasets.

**Figure 5.**
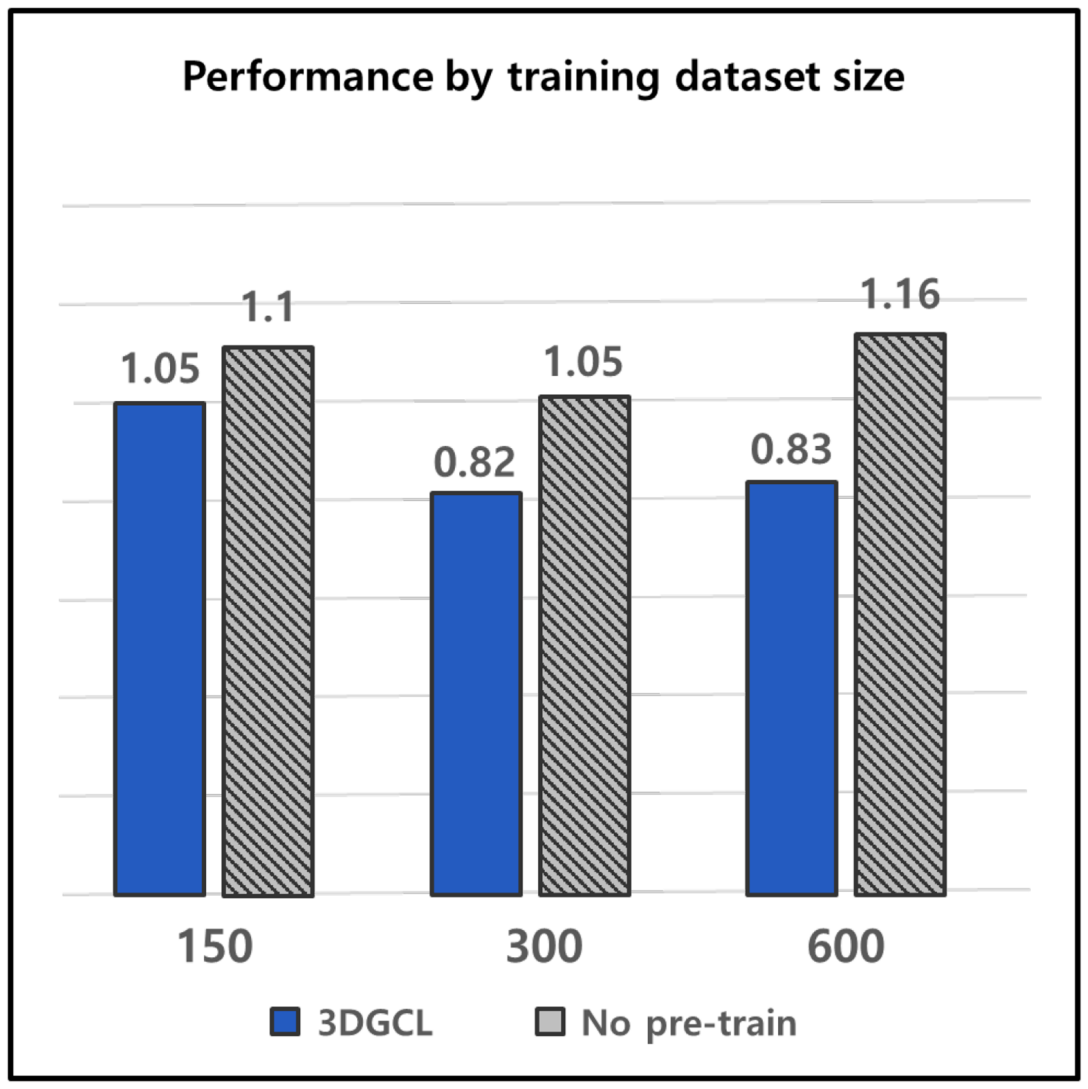
Comparison of performance difference by training dataset size

Then, we compare the model’s results with pretraining and the model without pretraining. The pretraining process was conducted using the whole ESOL dataset in the same way as in the above experiments. We evaluate the results based on RMSE and observe that our method consistently outperforms the model trained from scratch in all the reduced datasets, as shown in Fig. 5. The results indicate that our approach can be utilized in the drug field encountering annotation insufficiency to obtain significant effects.

## 6 Conclusion and further works

In this work, we first proposed a novel 3D–3D view Graph Contrastive Learning framework to address existing issues in SSL for molecular property prediction. Despite the prevailing notion that SSL requires hyper-scale resources, we present the possibility of small-scale SSL methods. Furthermore, we suggested a contrastive approach using randomization in a conformer pool that can learn 3D information in abundance while maintaining molecular semantics, unlike previous methods that could alter the property of the molecule. Comprehensive experiments show that our method outperforms existing self-supervised methods. We provide insight that it is proper to leverage 3D geometric information and domain-based knowledge in molecular property prediction. We also demonstrate that 3DGCL can be applied to the actual drug field undergoing annotation scarcity. We have shown remarkable performance using models and datasets, even on a small scale.

Despite the strong strengths, our model 3DGCL also has some weaknesses, such as focusing on only distance-based geometric information due to our 3D-based backbone framework. Another weakness is that the model needs to generate 3D information of molecules, which can slow the model training. If sufficient resources are supported, we will extend 3DGCL to further studies with a broader range of downstream tasks, along with hyperscale model capacity, and more diverse geometric information.

## Supplementary Material

btad371_Supplementary_Data

## References

[btad371-B1] Adams K , Pattanaik L, Coley CW. Learning 3D representations of molecular chirality with invariance to bond rotations. In: *International Conference on Learning Representations*, 2022.

[btad371-B2] Bemis GW , Murcko MA. The properties of known drugs. 1. Molecular frameworks. J Med Chem 1996;39:2887–93. 8709122 10.1021/jm9602928

[btad371-B3] Blum LC , Reymond J-L. 970 million drug-like small molecules for virtual screening in the chemical universe database gdb-13. J Am Chem Soc 2009;131:8732–3. 19505099 10.1021/ja902302h

[btad371-B4] Chen T , Kornblith S, Norouzi M et al A simple framework for contrastive learning of visual representations. In: *International Conference on Machine Learning*. PMLR, 2020, 1597–607.

[btad371-B5] Chithrananda S , Grand G, Ramsundar B. ChemBERTa: large-scale self-supervised pretraining for molecular property prediction. arXiv, 10.48550/arXiv.2010.09885, 19 October 2020, preprint: not peer reviewed.

[btad371-B6] Danel T , Spurek P, Tabor J et al Spatial graph convolutional networks. In: *International Conference on Neural Information Processing*, *Part V*. Springer, 2020, 668–675.

[btad371-B7] Delaney JS. ESOL: estimating aqueous solubility directly from molecular structure. J Chem Inf Comput Sci 2004;44:1000–5. 15154768 10.1021/ci034243x

[btad371-B8] Dillard L. 2021. Self-Supervised Learning for Molecular Property Prediction. Cambridge: Cambridge Open Engage.

[btad371-B9] Fang X , Liu L, Lei J et al Geometry-enhanced molecular representation learning for property prediction. Nat Mach Intell 2022;4:127–34.

[btad371-B10] Fey M , Lenssen JE. Fast graph representation learning with PyTorch geometric, arXiv, 10.48550/arXiv.1903.02428, 6 March 2019, preprint: not peer reviewed.

[btad371-B11] Ganea O , Pattanaik L, Coley C et al GeoMol: torsional geometric generation of molecular 3D conformer ensembles. Adv Neural Inf Process Syst 2021;34:13757–69.

[btad371-B12] Gasteiger J , Groß J, Günnemann S. Directional message passing for molecular graphs. In: *International Conference on Learning Representations*, 2020.

[btad371-B13] Gilmer J , Schoenholz SS, Riley PF et al Neural message passing for quantum chemistry. In: *International Conference on Machine Learning*, Sydney, Australia. PMLR, 2017, 1263–72.

[btad371-B14] Halgren TA. Merck molecular force field. I. Basis, form, scope, parameterization, and performance of MMFF94. J Comput Chem 1996;17:490–519.

[btad371-B15] He K , Fan H, Wu Y et al Momentum contrast for unsupervised visual representation learning. In: *Proceedings of the IEEE/CVF Conference on Computer Vision and Pattern Recognition*, 2020, 9729–38.

[btad371-B16] Hermosilla P , Ropinski T. Contrastive representation learning for 3D protein structures, arXiv, 10.48550/arXiv.2205.15675, 31 May 2022, preprint: not peer reviewed.

[btad371-B17] Hu W , Liu B, Gomes J et al Strategies for pre-training graph neural networks. In: *International Conference on Learning Representations*, 2020.

[btad371-B18] Kenton JDM-WC , Toutanova LK. BERT: pre-training of deep bidirectional transformers for language understanding. In: *Proceedings of NAACL-HLT*, Minneapolis, Minnesota, 2019, 4171–86.

[btad371-B19] Landrum G , Tosco P, Kelley B et al 2020. rdkit/rdkit: 2020_03_1 (q1 2020) release.

[btad371-B20] Li P , Wang J, Qiao Y et al An effective self-supervised framework for learning expressive molecular global representations to drug discovery. Brief Bioinf 2021;22:bbab109. 10.1093/bib/bbab109 33940598

[btad371-B21] Liu M , Luo Y, Wang L et al DIG: a turnkey library for diving into graph deep learning research. J Mach Learn Res 2021;22:1–9.

[btad371-B22] Liu S , Demirel MF, Liang Y. N-gram graph: simple unsupervised representation for graphs, with applications to molecules. Adv Neural Inf Process Syst 2019;32:8464–8467.

[btad371-B23] Liu S , Wang H, Liu W et al Pre-training molecular graph representation with 3D geometry. In: *International Conference on Learning Representations*, 2022a.

[btad371-B24] Liu Y , Wang L, Liu M et al Spherical message passing for 3D graph networks. In: *International Conference on Learning Representations*, 2022b.

[btad371-B25] Lu C , Liu Q, Wang C et al Molecular property prediction: a multilevel quantum interactions modeling perspective. In: *Proceedings of the AAAI Conference on Artificial Intelligence*, Honolulu, Hawaii, USA, Vol. 33, 2019, 1052–60.

[btad371-B26] Ma H , Bian Y, Rong Y et al Cross-dependent graph neural networks for molecular property prediction. Bioinformatics 2022;38:2003–9. 35094072 10.1093/bioinformatics/btac039

[btad371-B27] Mikolov T , Chen K, Corrado G et al Efficient estimation of word representations in vector space, arXiv, 10.48550/arXiv.1301.3781, 16 January 2013, preprint: not peer reviewed.

[btad371-B28] Mobley DL , Guthrie JP. Freesolv: a database of experimental and calculated hydration free energies, with input files. J Comput Aided Mol Des 2014;28:711–20. 24928188 10.1007/s10822-014-9747-x PMC4113415

[btad371-B29] Paszke A , Gross S, Massa F et al PyTorch: an imperative style, high-performance deep learning library. Adv Neural Inf Process Syst 2019;32.

[btad371-B30] Qiao Z , Welborn M, Anandkumar A et al OrbNet: deep learning for quantum chemistry using symmetry-adapted atomic-orbital features. J Chem Phys 2020;153:124111. 33003742 10.1063/5.0021955

[btad371-B31] Ramakrishnan R , Hartmann M, Tapavicza E et al Electronic spectra from TDDFT and machine learning in chemical space. J Chem Phys 2015;143:084111. 26328822 10.1063/1.4928757

[btad371-B32] Ramsundar B , Eastman P, Walters P et al 2019. Deep Learning for the Life Sciences. Sebastopol, CA: O’Reilly Media.

[btad371-B33] Rogers D , Hahn M. Extended-connectivity fingerprints. J Chem Inf Model 2010;50:742–54. PMID: 20426451. 20426451 10.1021/ci100050t

[btad371-B34] Rong Y , Bian Y, Xu T et al Self-supervised graph transformer on large-scale molecular data. Adv Neural Inf Process Syst 2020;33:12559–71.

[btad371-B35] Schütt K , Kindermans P-J, Sauceda Felix HE et al SchNet: a continuous-filter convolutional neural network for modeling quantum interactions. Adv Neural Inf Process Syst 2017;30.

[btad371-B36] Shi C , Luo S, Xu M et al Learning gradient fields for molecular conformation generation. In: *International Conference on Machine Learning*, 9558–68. PMLR, 2021.

[btad371-B37] Shui Z , Karypis G. Heterogeneous molecular graph neural networks for predicting molecule properties. In: *2020 IEEE International Conference on Data Mining (ICDM)*, Seoul, Korea, 492–500. IEEE, 2020.

[btad371-B38] Stärk H , Beaini D, Corso G et al 3D infomax improves GNNs for molecular property prediction. In: *International Conference on Machine Learning*, Baltimore, USA, 20479–502. PMLR, 2022.

[btad371-B39] Subramonian A. Motif-driven contrastive learning of graph representations. In: *Proceedings of the AAAI Conference on Artificial Intelligence*, Vol. 35, 2021, 15980–1.

[btad371-B40] Sun M , Xing J, Wang H et al MoCL: data-driven molecular fingerprint via knowledge-aware contrastive learning from molecular graph. In: *Proceedings of the 27th ACM SIGKDD Conference on Knowledge Discovery & Data Mining*, 3585–94, 2021. 10.1145/3447548.3467186 PMC9105980 35571558

[btad371-B41] Unke OT , Meuwly M. PhysNet: a neural network for predicting energies, forces, dipole moments, and partial charges. J Chem Theory Comput 2019;15:3678–93. PMID: 31042390. 31042390 10.1021/acs.jctc.9b00181

[btad371-B42] Vaswani A , Shazeer N, Parmar N et al Attention is all you need. Adv Neural Inf Process Syst 2017;30.

[btad371-B43] Wang S , Guo Y, Wang Y et al SMILES-BERT: large scale unsupervised pre-training for molecular property prediction. In: *Proceedings of the 10th ACM International Conference on Bioinformatics, Computational Biology and Health Informatics, New York, USA*, 429–436, 2019.

[btad371-B44] Wang Y , Wang J, Cao Z et al Molecular contrastive learning of representations via graph neural networks. Nat Mach Intell 2022;4:279–87.

[btad371-B45] Weininger D. SMILES, a chemical language and information system. 1. Introduction to methodology and encoding rules. J Chem Inf Comput Sci 1988;28:31–6.

[btad371-B46] Wu Z , Ramsundar B, Feinberg EN et al MoleculeNet: a benchmark for molecular machine learning. Chem Sci 2018;9:513–30. 29629118 10.1039/c7sc02664a PMC5868307

[btad371-B47] Xiong Z , Wang D, Liu X et al Pushing the boundaries of molecular representation for drug discovery with the graph attention mechanism. J Med Chem 2020;63:8749–60. 31408336 10.1021/acs.jmedchem.9b00959

[btad371-B48] Yang K , Swanson K, Jin W et al Analyzing learned molecular representations for property prediction. J Chem Inf Model 2019;59:3370–88. PMID: 31361484. 31361484 10.1021/acs.jcim.9b00237 PMC6727618

[btad371-B49] You Y , Chen T, Sui Y et al Graph contrastive learning with augmentations. Adv Neural Inf Process Syst 2020;33:5812–23.

[btad371-B50] Zhou G , Gao Z, Ding Q et al Uni-Mol: a universal 3D molecular representation learning framework. In: *International Conference on Learning Representations*, Kigali, Rwanda, 2023.

[btad371-B51] Zhu Y , Chen D, Du Y et al Featurizations matter: a multiview contrastive learning approach to molecular pretraining. In: *ICML 2022 2nd AI for Science Workshop*, Baltimore, USA, 2022.

